# Exploiting Natural Cross-reactivity between Human Immunodeficiency Virus (HIV)-1 p17 Protein and Anti-gp41 2F5 Antibody to Induce HIV-1 Neutralizing Responses *In Vivo*

**DOI:** 10.3389/fimmu.2017.00770

**Published:** 2017-06-30

**Authors:** Bernard Verrier, Stéphane Paul, Céline Terrat, Liza Bastide, Agathe Ensinas, Capucine Phelip, Blandine Chanut, Laura Bulens-Grassigny, Fabienne Jospin, Christophe Guillon

**Affiliations:** ^1^Colloidal Vectors and Tissue Transport, UMR5305, Institut de Biologie et Chimie des Protéines, Université de Lyon, CNRS, Lyon, France; ^2^Groupe sur l’Immunité des Muqueuses et Agents Pathogènes, EA3064, Faculté de Médecine Jacques Lisfranc, Université de Lyon, Saint-Etienne, France; ^3^Retroviruses and Structural Biochemistry, UMR5086, Institut de Biologie et Chimie des Protéines, Université de Lyon, CNRS, Lyon, France

**Keywords:** human immunodeficiency virus-1, gp41, p17, neutralizing antibodies, cross-reactivity, antigen engineering

## Abstract

Anti-p17 antibodies are able to neutralize human immunodeficiency virus (HIV) entry in a mouse model. In this study, we identified a region of sequence similarity between the epitopes of anti-p17 neutralizing antibodies and anti-gp41 neutralizing 2F5 antibody and verified cross-reactivity between p17 and 2F5 *in vitro*. The p17 sequence was modified to increase sequence identity between the p17 and 2F5 epitopes, which resulted in enhanced cross-reactivity *in vitro*. Immunogenicity of wild-type and modified p17 was characterized in a rabbit model. Both wild-type and mutated p17 induced anti-gp41 responses in rabbits; sera from these animals reacted with gp41 from different HIV clades. Moreover, introduction of the 2F5 sequence in p17 resulted in induction of antibodies with partially neutralizing activity. Based upon these data, we suggest that the natural cross-reactivity between HIV-1 p17 protein and 2F5 antibody can be exploited to induce antibodies with neutralizing activity in an animal model.

## Introduction

Despite the high variability of human immunodeficiency virus (HIV), approximately 15–30% of infected patients develop broadly neutralizing antibodies (bnAbs) able to neutralize entry of the majority of HIV strains (1–3). In non-human primates, passive administration of such bnAbs protects animals from infection (4). Although several bnAbs have been characterized (2, 5), the method by which to induce such antibodies by vaccination remains an important issue (3, 6–9). Preclinical studies in various models suggest that sequential immunizations, with multiple injections and/or multiple antigens, may be one way to elicit bnAbs (10–12). However, current vaccine strategies have not yet been able to induce high bnAbs titers (13).

Another important target for HIV vaccination is the Gag polyprotein. The simian immunodeficiency virus Gag capsid subunit undergoes strong structural constraints, which limit its variability and, therefore, its ability to accumulate mutations in order to elude the host immune system (14). Indeed, broad cytotoxic T-lymphocyte (CTL) responses targeting Gag are associated with a low viral load in HIV-infected people (15–17), and induction of CTL responses against HIV-1 Gag could lead to elimination of HIV-1-infected cells and aid in control of viral dissemination (18). Moreover, antibody responses against the p17 subunit of Gag could block the pathogenic effects of extracellular p17 (19, 20).

As Gag proteins are not exposed on the virion surface, few studies have systematically explored the antibody responses induced by immunization with Gag proteins and their potential to inhibit HIV infection. However, cross-reactivity between p17 and gp41 can occur (21), and viral entry can be neutralized by antibodies against p17 (22). The p17 sequence, ELDKWRK, is part of the epitope of an anti-p17 antibody that neutralizes HIV entry (22), and a bnAb, 2F5, directed against the HIV gp41 envelope subunit, was identified in an infected individual (23, 24). Interestingly, this 2F5 antibody recognizes an epitope with the sequence ELDKWAS in the membrane proximal external region (MPER) of gp41 (24). This pentapeptide, ELDKW, is shared by HIV-1 p17 and the MPER of HIV-1 gp41, and is recognized by two neutralizing antibodies targeting p17 and gp41. However, the structure of the 2F5 antibody in complex with its epitope shows the AS residues of the 2F5 epitope ELDKWAS involved in the antibody/peptide interaction and in the conformation of the epitope (25).

In the present study, we demonstrated cross-reactivity between the anti-gp41 2F5 antibody and the wild-type p17 protein. We then introduced the entire ELDKWAS 2F5 epitope in the context of the ELDKW pentapeptide into p17 and evaluated cross-recognition *in vitro*. Finally, preliminary immunization experiments in an animal model confirmed the ability of p17 to induce anti-gp41 immune responses with neutralizing activities, which are improved by mutating p17.

## Materials and Methods

### p17 Constructs

**Figure 1:**
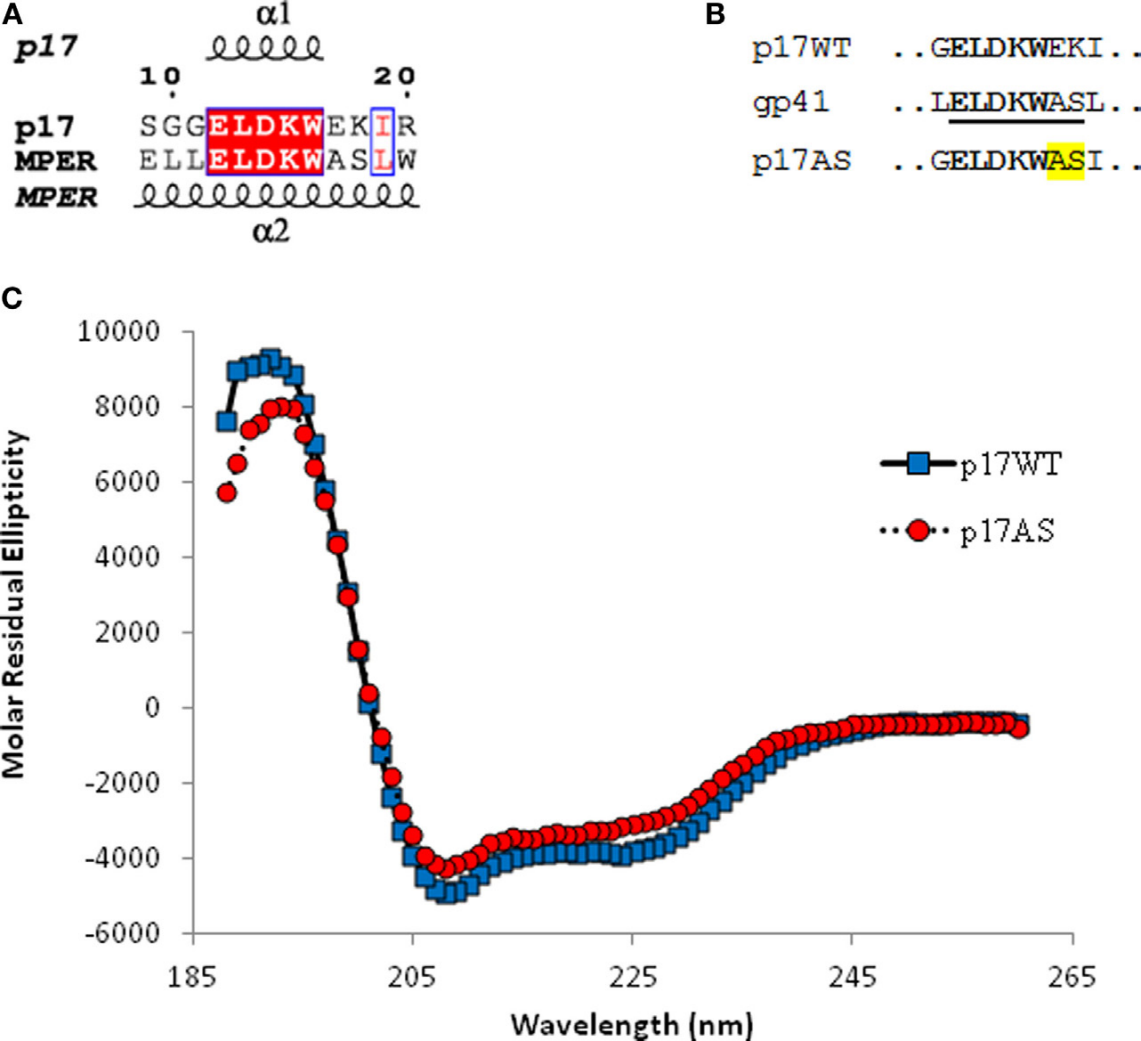
Design and characterization of p17AS mutant. **(A)** Partial alignment and structural environment of the amino acid sequences of p17 residues 9–20 and the membrane proximal external region (MPER) region of gp41 residues 661–672 [numbering according to the pNL4-3 Gag polyprotein and HXB2 Env sequences, respectively (27)]. Identity and similarity are depicted by red and empty boxes, respectively. Secondary structures are extracted from PDB ID 1HIW (28) for p17 and PDB ID 5IQ7 (29) for MPER. Rendering was performed using the ESPript server http://espript.ibcp.fr (30); **(B)** partial alignment of the amino acid sequences of p17WT and gp41 around the shared pentapeptide ELDKW (in bold) with the mutant p17 (p17AS). Underlined is the 2F5 epitope in gp41. The p17AS mutation is highlighted in yellow; **(C)** far UV circular dichroism spectra obtained for wild-type (blue squares) or p17AS mutant (red circles).

Amino acid sequences of the various constructs are described in Figures 1A,B. The wild-type gene (p17WT) from the pNL4-3.Luc.R^−^.E^−^ plasmid (26) was amplified by PCR in-frame with a C-terminal 6 × His tag. The p17AS mutant was obtained from the p17WT coding sequence using Phusion polymerase (Finnzymes) and primers p17AS-R (5′-GATAAATGGGCAAGCATTCGGTTAAG-3′) and p17AS-F (5′-CTTAACCGAATGCTTGCCCATTTATC-3′). The p17 constructs were inserted into the pRSET-B plasmid (Invitrogen) using *Nde*I/*Eco*RI restriction sites.

### Protein Expression, Purification, and Analysis

The p17 constructs were expressed in BL21(DE3)pLys competent cells (Lucigen). Lysogeny broth (250 ml) supplemented with 25 µg/ml ampicillin were seeded with an overnight preculture and incubated at 37°C with agitation. When OD_600nm_ ≈ 0.4, protein expression was induced with 2 mM isopropyl-β-d-1-thiogalactopyranoside (Euromedex) and incubated for 5 h at 37°C. Cultures were then centrifuged at 6,000 × *g* for 5 min, and pellets were stored overnight at −20°C.

Bacterial lysis and protein purification using Ni-TED resin (Macherey-Nagel) were performed as previously described (31), except lysis was performed by sonication of the pellet for4× 1 min/g with a Sonifier 250 sonicator (Brandson).

Following protein purity analysis with sodium dodecyl sulfate polyacrylamide gel electrophoresis (SDS-PAGE), elution fractions were pooled, dialyzed against 50 mM 2-(*N*-morpholino)ethanesulfonic acid (MES), pH 6, overnight at 4°C, and concentrated to 1 mg/ml according to the absorbance of the protein solution at 280 nm, using a Vivaspin-PES 10 kDa (Sartorius).

Circular dichroism spectra were recorded and processed using a Chirascan Dichrometer (Applied Photophysics) as described previously (32), with 150 µl of protein at 0.2 mg/ml in 50 mM MES pH 6.

### Enzyme-Linked Immunosorbent Assay (ELISA) Cross-reactivity

Anti-p17 mouse monoclonal antibody 8E7A3 (isotype IgG1κ) was obtained after immunization of mice with HIV Gag (not shown). Anti-gp41 2F5 human antibody (Polymun Scientific) and isotypic controls (anti-gp120 F105 and anti-Tat 11H6H1 for 2F5 and anti-p17, respectively) have been described elsewhere (24, 33, 34). In order to evaluate cross-reactivity by ELISA, 100 ng/well p17WT or p17AS protein in 1× phosphate-buffered saline (PBS) were coated onto 96-well plates (MaxiSorb, Nunc) overnight at 25°C. Wells were saturated with 1× PBS-10% horse serum (Thermo Fisher Scientific), incubated for 1 h at 37°C, washed with 1× PBS-0.05% Tween 20 (Sigma-Aldrich), and then incubated with serial dilutions of the antibodies in duplicate in PBS-10% horse serum for 1 h at 37°C. After washing, wells were incubated with 100 µl horseradish peroxidase (HRP)-conjugated anti-human or anti-mouse IgG (Jackson Immunoresearch) diluted to 0.5 µg/ml in PBS-Tween and visualized using BD-OptiEA reagent (BD Bioscience). OD values were measured at 450 nm with a reference at 620 nm, using a Bio-Rad plate reader.

### Antigen Formulation and Rabbit Immunization

The p17WT or mutant p17AS, diluted to 50 µg/ml in 1× PBS, were adsorbed on poly-lactic acid (PLA) nanoparticles for 2 h at 25°C. Adsorption yield was calculated as previously described (35). After washing, antigen/PLA complexes were resuspended in 1× PBS to a final adsorbed protein concentration of 0.2 mg/ml according to the calculated yield. Formulations were stored at 4°C overnight before immunization the following day and remained homogeneous during all processes as verified by dynamic light scattering (data not shown).

Two groups of three rabbits (20-week-old New Zealand White female rabbits weighting 2.5 kg at the start of the experiment, Charles River Laboratories) were immunized subcutaneously with 100 µg of formulated p17WT or p17AS proteins. Animals were immunized at day (d) 0, d28, and d63. Prior to each immunization and at d14, d42, and d77, 5 ml of sera were collected from each animal. Animals were sacrificed and exsanguinated at d98 when 3.5–5 ml of serum were collected depending on the animals. Animals were housed and manipulated according to French standard regulatory ethical and welfare guidelines of the PLEXAN, Saint-Étienne (authorization no 42-218-0801) and approved by the relevant ethics committee (Comité d’Ethique en Expérimentation Animale de la Loire (CEEAL-UJM n 98), Faculté de Médecine Jacques Lisfranc, Saint-Étienne; reference 00811.02).

### Immunization Follow-up

Anti-p17 IgG titers were quantified by ELISA, with the immunogen coated onto plates overnight at 100 ng/well in PBS. After saturation and washes (described above in Section “Antigen Formulation and Rabbit Immunization”), sera were incubated at different concentrations for 1 h at 37°C and visualized using a HRP-conjugated anti-rabbit IgG Fc fragment (P.A.R.I.S.) at 0.1 µg/ml. Anti-gp41 titers were quantified under the same conditions, with wells coated with 100 ng/well of recombinant HXB2 gp41 protein. Titers were measured as the reciprocal of the first dilution giving a negative signal (<0.2 OD).

Sera from d98 diluted to 1:5,000 in PBS were evaluated for their reactivity against a panel of recombinant gp41 fragments, which have been described previously (36, 37). Briefly, the proteins were coated at a concentration of 100 ng/well overnight. After saturation and washes (described above in Section “Enzyme-Linked Immunosorbent Assay (ELISA) cross-reactivity”), sera were incubated for 1 h at 37°C before being visualized with HRP-conjugated anti-rabbit IgG Fc fragment (Bethyl), as previously described (35). The immunizing antigen and an aspecific control protein (bovine serum albumin) (Thermo Fisher Scientific) were used as positive and negative controls, respectively.

### Neutralization Assay

IgG were purified from animal samples at d98 using the protein G HP SpinTrap/Ab SpinTrap kit (GE Healthcare), and IgG concentrations were measured using an IgG ELISA (37). Purified IgG did not show autoreactivity when tested at 20 µg/ml against 293T or Hep2 cells (data not shown). The neutralization assay was performed using T-cell line-adapted strain LAI (clade B) or primary isolates of clade A (92UG029), B (SF162, 92US660), and D (92UG001). Neutralizing activities of purified IgG were measured as described previously (37) on SupT1 cells or peripheral blood mononuclear cells (PBMCs) for the LAI strain and the primary isolates, respectively, in duplicate. PBMCs were isolated using standard Ficoll purification before being activated with phytohemagglutinin (1 µg/10^6^ cells) for 48 h. Purified rabbit IgG (50 µl, concentrated to 100 µg/ml) was incubated with 50 µl virus for 3 h at 37°C prior to being added to 100 µl cells (3.10^6^ cells/ml) overnight. Cells where then washed with culture medium and incubated for 5 or 7 days (PBMCs and SupT1, respectively) before the supernatant was harvested. The concentration of p24 in the supernatant was determined using the p24 kit (InfYnity). Neutralization percentage was calculated as the reduction of p24 production compared to a control without rabbit IgG. Statistical analysis was performed using a Mann–Whitney *U*-test or a one-way analysis of variance (ANOVA) with a Bonferroni post-test (GraphPad 7.0), combining all individual data obtained for each group.

## Results

### Evidence of Cross-Recognition of p17 by 2F5 Antibody

Alignment of the p17 N-terminal region with the MPER of gp41 containing the 2F5 epitope reveals the presence of a common pentapeptide, ELDKW, located in a similar α-helical environment (Figure 1A). Based upon this data, we introduced mutations into p17WT to re-create the complete 2F5 cognate epitope, ELDKWAS, in p17 (Figure 1B). This mutation, termed p17AS, did not impair the structure of the p17 protein, as verified by circular dichroism (Figure 1C).

**Figure 2:**
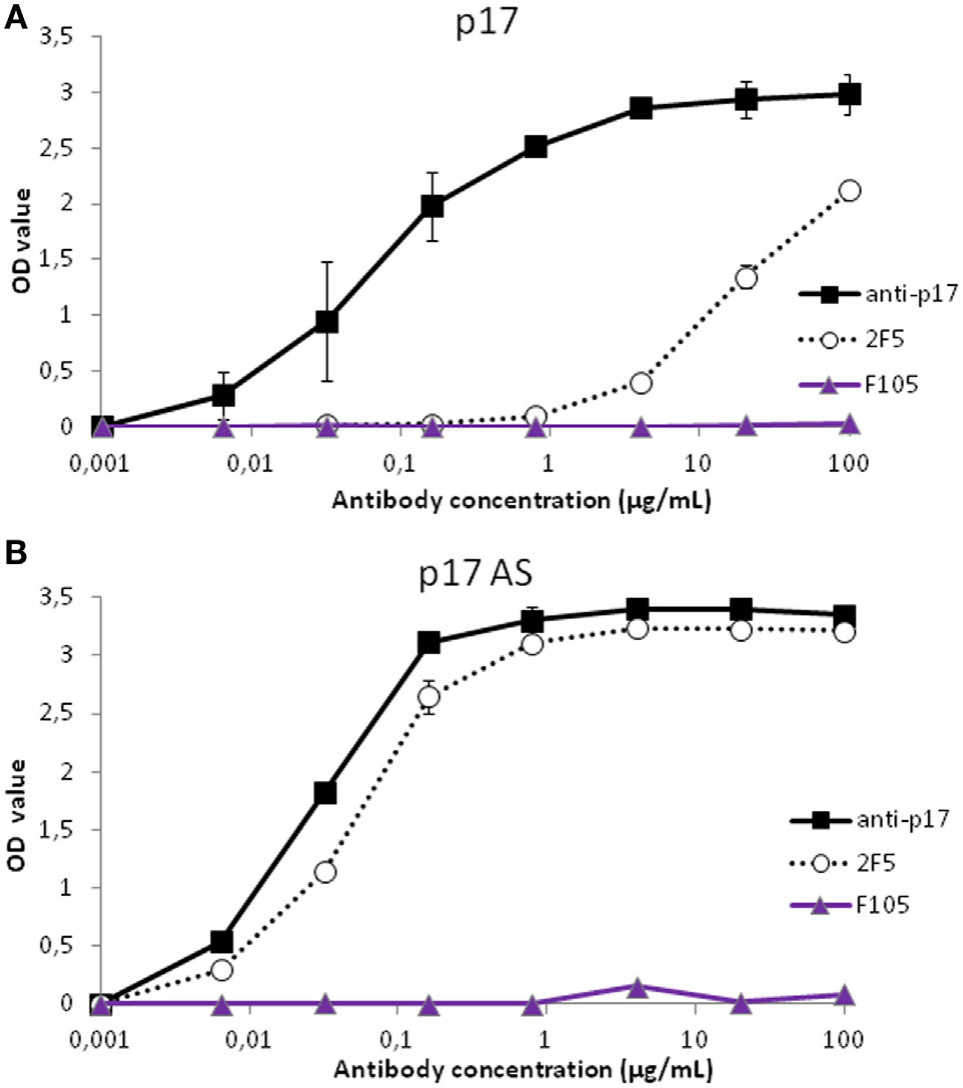
bnAb 2F5 cross-reacts with p17 *in vitro*. **(A,B)** Reactivity of p17WT **(A)** or p17AS mutant **(B)** against anti-p17 (black squares, plain lines) and anti-gp41 2F5 (white circles, dotted line) antibodies. Irrelevant antibody binding (F105 antibody) is displayed with purple triangles and lines. Values represent the mean value of four different experiments in duplicate, and error bars represent the SD of the same measures.

Both p17WT and p17AS reacted similarly with the anti-p17 antibody (Figures 2A,B). The p17WT protein was detected with the anti-gp41 2F5 antibody (the cognate sequence contains ELDKW), but not with the isotype control antibodies, anti-gp120 F105 (Figure 2A) and anti-Tat (data not shown), demonstrating a specific but low cross-reactivity between p17 and the anti-gp41 2F5 antibody. The p17AS mutation resulted in an increase in reactivity of 2F5 for p17AS compared to p17WT *in vitro*, as demonstrated by an approximately 2 log decrease in the apparent half maximal effective concentration (EC_50_) (Figure 2B). The EC_50_ values for 2F5 against p17AS were in the same range of the EC_50_ for a specific anti-p17 antibody against its natural target, p17 (Figure 2B).

Thus, the gp41-specific 2F5 bnAb cross-reacted with the p17 Gag subunit. Introduction of the 2F5 epitope into p17 (p17AS) increased cross-recognition of p17 by the 2F5 antibody, confirming that the structural context of this region of p17 was favorable for cross-recognition.

### Cross-Clade Anti-gp41 Responses in Rabbits Induced by p17

Immunogenicity of p17WT and p17AS was evaluated in rabbits. Groups of three rabbits were immunized at d0, d28, and d63 with 100 µg of protein formulated on PLA particles, with serum samples taken every 2–3 weeks.

**Figure 3:**
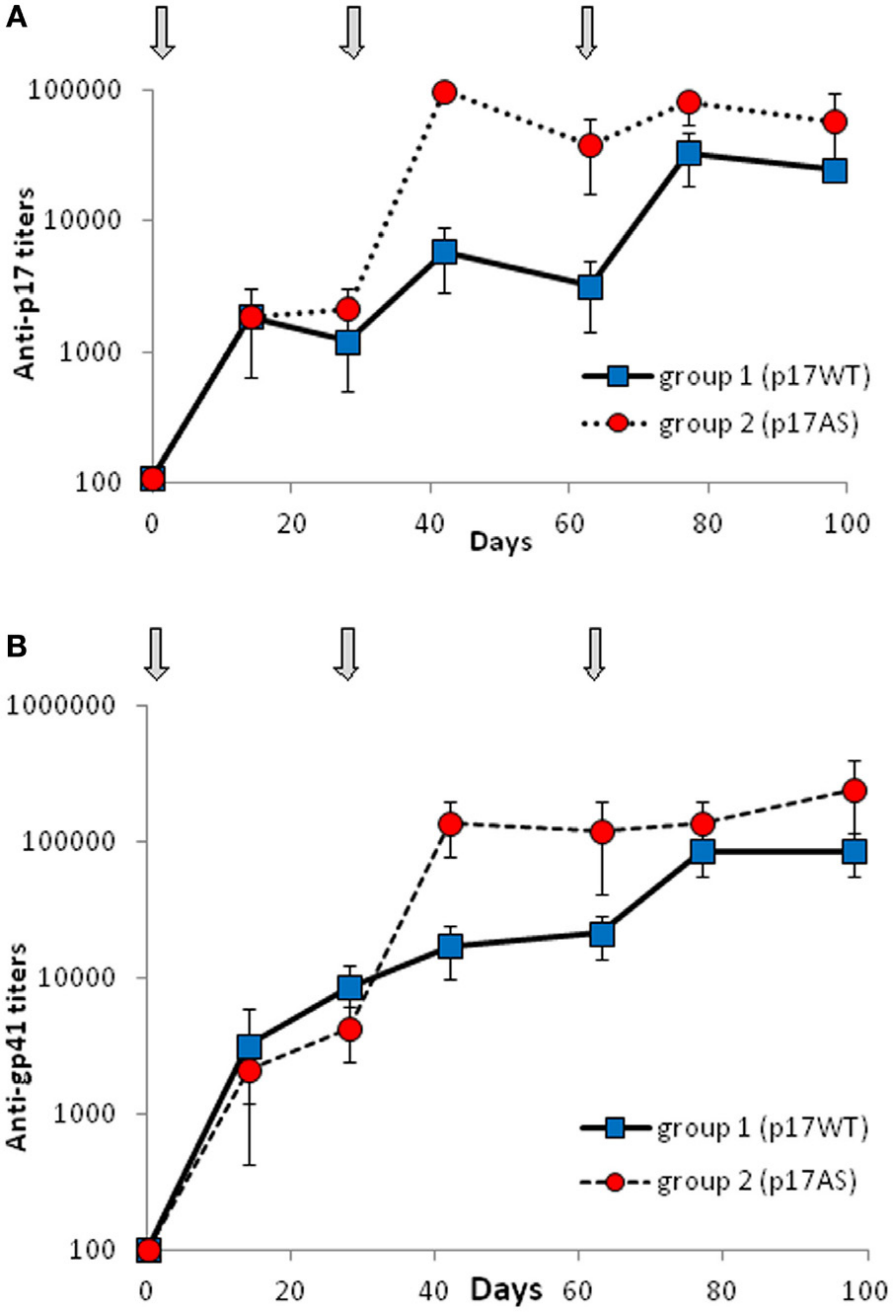
Immunization follow-up. **(A,B)** Serum titration of New Zealand White rabbits immunized by p17WT (blue squares, plain line) or p17AS (red circles, dotted line) against **(A)** the immunizing p17 protein and **(B)** recombinant gp41 protein (HXB2 strain). Gray arrows indicate the dates of immunizations. Each value represents the mean of the titers of sera from the three rabbits of each group, measured individually in duplicate. Error bars represent the SD of the same measures.

Antibody titers against the immunogen were estimated for each sampling point as the first dilution yielding a negative signal, and demonstrated elicitation of a high anti-p17 antibody titer (~10^5^) by both immunogens (Figure 3A). Anti-p17 antibodies appeared to be present earlier in animals immunized with p17AS compared to p17WT, but the differences in titers at d98 were not statistically significant (Mann–Whitney test, *p* = 0.4).

To confirm that p17 could induce an anti-gp41 response, the same sera were titrated against clade B gp41 (HXB2). Anti-gp41 titers showed the same profile as anti-p17 titers, reaching at least 10^5^ at d98 (Figure 3B), demonstrating that immunization with p17 can indeed induce strong titers of antibodies able to recognize gp41. At d98, there was no statistically significant difference between the two groups (Mann–Whitney test, *p* = 0.4), suggesting that amounts of induced anti-gp41 antibodies were not quantitatively higher when immunized with the p17AS mutant.

**Figure 4:**
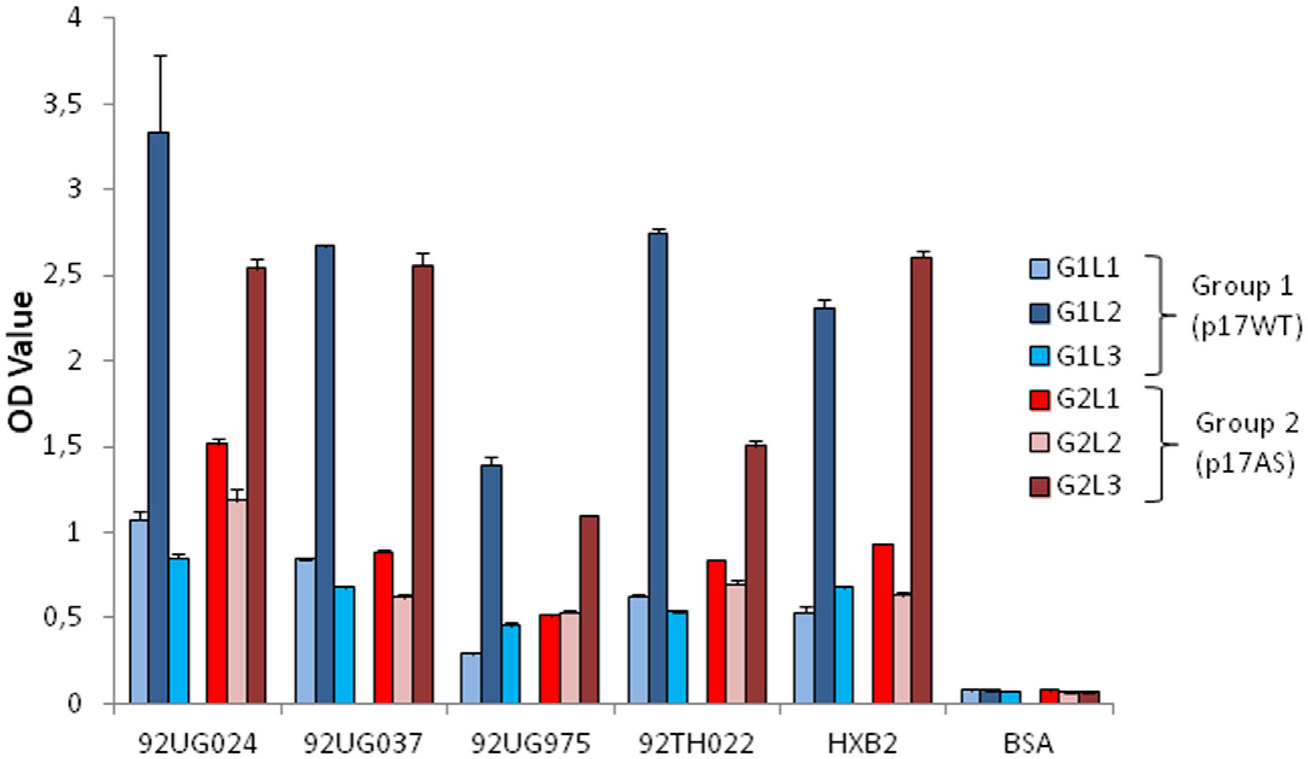
Cross-clade reactivity of p17-induced anti-gp41 antibody responses. Recognition of recombinant gp41 fragments from different subtypes by the sera at d98 of each rabbit immunized by p17WT (blue taints) or p17AS (red taints), diluted 1/5,000. Each value represents the mean of the OD value measured individually in duplicate. Error bars represent the SD of the same measures. BSA is the negative protein control.

The cross-clade reactivity of these anti-gp41 antibodies was assessed through analysis of the sera for reactivity against gp41 fragments from different HIV-1 clades. As expected from anti-gp41 titer results, the serum from each animal recognized gp41 from the clade B strain (HXB2) (Figure 4), as well as strains from clades A, AE, D, and G (92UG037, 92UG024, 92TH022, and 92UG975, respectively) (Figure 4). These data indicate that the antibody response induced by p17 is able to cross-react with Env isolates from different clades around the 2F5 epitope. There was no significant difference in reactivity against the different proteins when immunized with p17WT or p17AS (Figure 4). Notably, the clade G strain, 92UG975, which exhibited the lowest signal (Figure 4), contains two mutations in the 2F5 epitope (ALDKWTS) compared to the clade B HXB2 strain used as a positive control (ELDKWAS) (27). As both mutations involve residues that participate in the 2F5/epitope interaction (25), this result suggests that the cross-reactivity of these sera targets the 2F5 epitope. Thus, immunizing rabbits with p17WT or p17AS would induce cross-clade-reactive anti-gp41 antibodies.

### Antibodies Induced by Immunization with p17AS Show Neutralizing Activities

**Figure 5:**
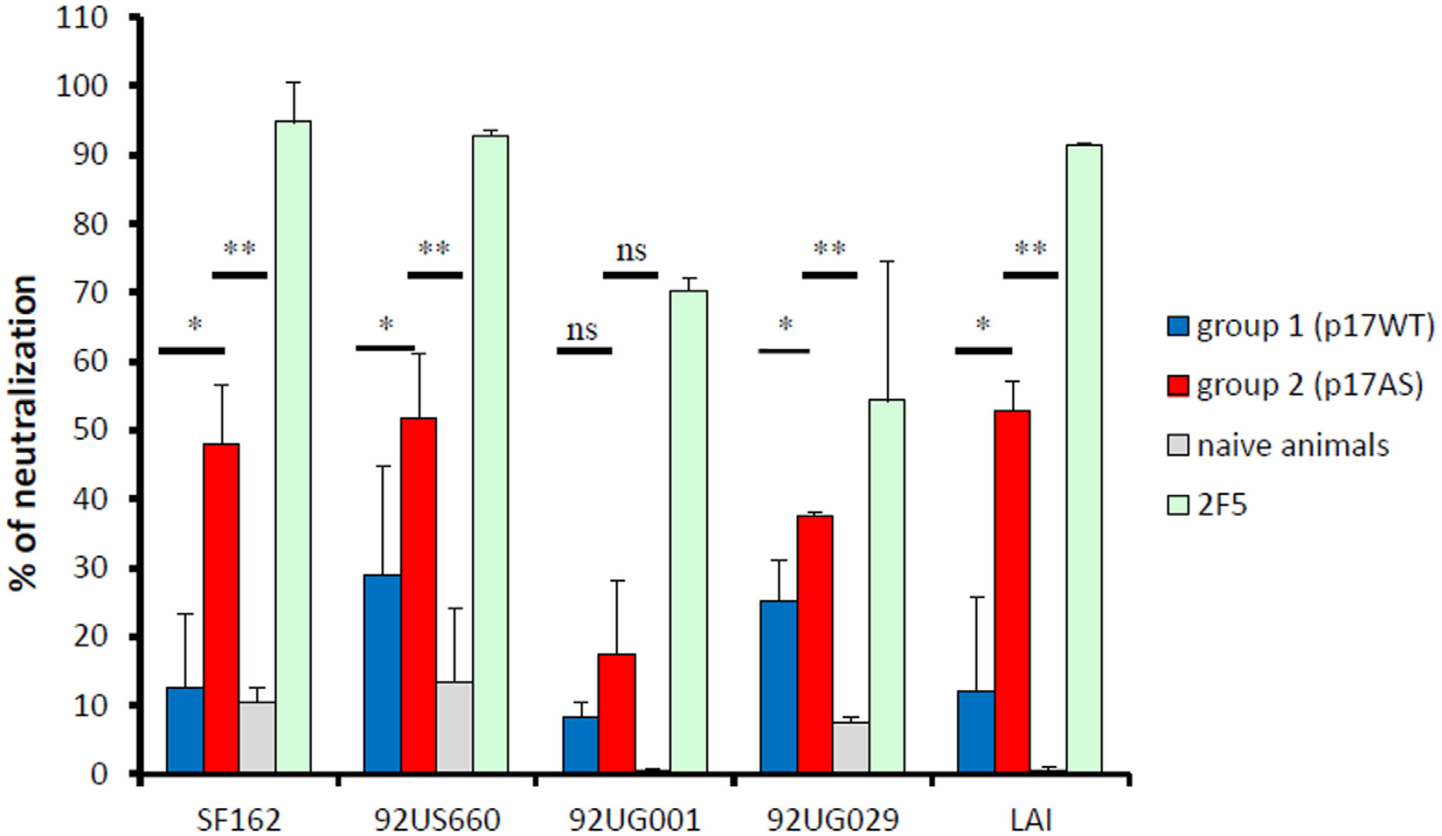
Neutralizing activity of p17-induced anti-gp41 antibody responses. Neutralizing efficacy of purified IgG at d98 from animals immunized with p17WT (blue bars) and p17AS (red bars) against viral particles pseudotypes using envelop proteins of various isolates. Reference antibody 2F5 (green bars) and sera from non-immunized animals (gray bars) were used as positive and negative controls, respectively. Each value represents the mean of the neutralizing efficacy of purified IgG from the three rabbits of each group, measured individually in duplicate. Error bars represent the SD of the same measures. Stars indicate significant differences between the two groups in neutralizing activity against the isolate.

The sera of animals immunized with p17AS significantly neutralized the infectivity of viruses pseudotyped with the Env protein of the LAI strain compared to p17WT or pre-bled animals (ANOVA, *p* < 0.05 and *p* < 0.01, respectively) (Figure 5). This also held true for viruses pseudotyped with envelopes of primary isolates (Figure 5): p17AS-induced antibody responses able to neutralize Tier 1, clade B strains (SF162, 92US660), and one Tier 2/3, clade A isolate (92UG029), with significant neutralizing activities for animals immunized with the modified p17AS protein against these strains compared to p17WT or pre-bled animals (*p* < 0.05 and *p* < 0.01, respectively), reaching up to 60% of neutralization (Figure 5). Infectivity of Tier 2/3, clade D isolate 92UG001, was only marginally affected by sera from both groups, with no significant difference between the two groups of animals (Figure 5, *p* > 0.5).

Thus, although complete neutralization was not achieved, sera from p17AS-immunized animals had neutralizing activity against Tier 1 and Tier 2/3 isolates.

## Discussion

In this study, we demonstrated that the gp41-specific 2F5 bnAb cross-reacted with the p17 Gag subunit. We also found that introduction of the full-length 2F5 epitope in p17 (mutant p17AS) increased this cross-reactivity *in vitro*. Furthermore, sera obtained after immunization with p17AS neutralized Tier 1 and Tier 2/3 isolates from different clades. Neutralizing responses were detectable after three immunizations with p17AS in our study, whereas rabbits immunized with trimeric gp140 envelope proteins have detectable neutralizing responses following a fourth immunization (10). This discrepancy could be due to the heavy glycosylation pattern of gp140 which may have reduced the elicitation of anti-gp140 neutralizing antibody responses (38), while our p17 antigens were not glycosylated.

In order to avoid unnecessary animal experiments, we performed a proof-of-concept study with a small number of animals, which can now be extended to a larger cohort of animals to improve statistical power. Despite the limited number of animals, our present study clearly demonstrated that p17WT and p17AS elicited antibodies in rabbits that recognize gp41 proteins from different clades and, for p17AS, possess partial neutralizing activity. Noteworthy, these gp41-reactive, cross-clade antibodies with partial neutralizing activity were obtained without the use of Env as an immunogen. This could explain the lower neutralizing activity we observed for p17AS-induced antibodies compared to other preclinical studies in which rabbits were immunized with gp140 (10). Our results suggest that cross-clade-reactive neutralizing anti-gp41 antibodies could be developed using this p17-targeting strategy. The next step is to evaluate the dose–response efficacy of neutralizing antibodies induced by p17 constructs against a larger panel of viruses (10, 39). The quality of the antibodies induced by p17 constructs for other antiviral activities, such as antibody-dependent cell-mediated cytotoxicity or virus inhibition will also be assessed.

Although some exceptions occur (40), most bnAb recognize their epitope in the lipid bilayer area of the viral particle (41). Interestingly, p17 is naturally associated with the lipid bilayer during viral replication due to the presence of an N-terminal myristoyl group (42). This location brings p17 ELDKW close to the lipid bilayer, which may enhance the induction of neutralizing antibodies. As mentioned previously, this point could not be verified as the p17 constructs were overexpressed in a bacterial system lacking natural myristoylation activity. Therefore, evaluation of cross-reactivity and immunogenicity of myristoylated forms of our p17-derived constructs is being considered.

Poly-lactic acid particles were used as an adjuvant as adsorption of the antigen is simple and reproducible, and such formulations preserve antigenicity and immunogenicity of the adsorbed antigens (43), which can also be oriented by the encapsulation of TLR ligands within the particles (44). This versatility allows for straightforward examination of the extent to which engineering of the p17 antigen, such as with the p17AS mutant, helps increase the breadth or magnitude of an induced anti-gp41 response (45). In parallel, structural information regarding interaction of the 2F5 antibody with the p17 constructs has been initiated to assist in structure-based engineering of the p17 antigen. Indeed, if an increase in sequence homology in p17AS increases the reactivity of 2F5 against p17, induced sera would be less reactive against isolates that contain a mutated sequence of the 2F5 epitope. Thus, increasing sequence homology while maintaining the structural environment of the 2F5 epitope in p17 will be the next step in optimization of a potent p17-derived antigen.

We demonstrated that sera obtained after immunization with p17AS neutralized infection by Tier 1 and Tier 2/3 isolates *in vitro*. Thus, p17 antigen can be used in an epitope scaffolding approach (46, 47), with the advantage of being a carrier that possesses vaccinal properties. As p17 induces strong CTL responses, such an approach would result in generation of a multivalent candidate antigen inducing both anti-Gag and anti-gp41 responses. Similarly, epitope scaffolding using Gag p24 subunit as a carrier for Env epitopes has been suggested (48, 49). These previous studies are based solely on bio-computational predictions and biophysical/biochemical validations. In contrast, our approach takes advantage of the existing cross-reactivity of p17 with bnAb 2F5, and this cross-reactivity resulted in induction of anti-Env antibodies with some neutralizing efficacy in an animal model.

In conclusion, we have confirmed the natural cross-reactivity between HIV-1 p17 protein and gp41-specific 2F5 antibody which was suspected from previous studies (22). We have established the proof-of-concept that this property can be exploited to induce anti-HIV-1 neutralizing antibody responses by validating several points: first, the HIV-1 p17 protein induces anti-gp41 responses in rabbits. Second, p17 can be modified to increase its recognition by anti-gp41 antibodies *in vitro*. Finally, such a modified p17 induces antibodies with neutralizing activity in a rabbit model *in vivo*. Thus, our study paves the way for rational design of multifunctional antigens derived from p17 to induce both protective responses against Gag and neutralizing antibodies against gp41 as part of a vaccine formulation against HIV-1 infection.

## Ethics Statement

Animals were housed and manipulated according to French standard regulatory ethical and welfare guidelines of the PLEXAN, Saint-Étienne (authorization no 42-218-0801), and approved bythe relevant ethics committee (Comité d’Ethique en Expérimentation Animale de la Loire (CEEAL-UJM n 98), Faculté de Médecine Jacques Lisfranc, Saint-Étienne; reference 00811.02).

## Author Contributions

BV, SP, and CG designed the study. CG, LB-G, and LB produced the antigens and characterized *in vitro* cross-reactivities. CP and CT formulated the antigens. FJ, AE, and BC immunized the animals and performed neutralizing studies. CT and AE performed the ELISA with animal sera. BV, SP, CT, and CG analyzed the data. All authors participated to the writing of the manuscript.

## Conflict of Interest Statement

The authors declare that the research was conducted in the absence of any commercial or financial relationships that could be construed as a potential conflict of interest.
